# Night-Blindness

**Published:** 1899-11-25

**Authors:** Clements Hailes

**Affiliations:** Assistant-Surgeon to the Bristol Eye Hospital.


### Hospital Clinics and Medical Progress.

### NIGHT-BLINDNESS.

By Clements Hailes, M.D., F.R.C.S., C.M. (Edin.), Assistant-Surgeon to the Bristol Eye Hospital.

Night-blindness, called also nyctalopia or hemeralopia, is a functinal disorder of the visual acuity causing great inconvenience, and often producing a state of mental panic in the unfortunate person who is suddenly affected by it. An individual still retaining his normal acuity of vision in the daylight suddenly notices that his sight has failed when twilight comes on; he passes an anxious evening, each endeavour of his to prove to himself that it is a mere passing fancy being contradicted by some fresh indication that his vision has undergone some change for the worse, drifts on to a wakeful night of suspense, eventually falling off to sleep, and, when daylight comes, awaking with the dread still on him, to find that with the morning his sight has returned, and he can see as well as ever. However, when the twilight comes again, or should he during the day go into a dimly-lighted apartment, he receives a fresh disappointment, and finds his sight is failing him again. This phenomena often happens to young sailors voyaging through the equatorial zone when they are out of reach of medical assistance, and knowing as they do that their livelihoods depend on the retention of their sight, and that no skilled adviser is at hand, they suffer considerably in their spirits as well; and depression of mind only increases the trouble, though sometimes old and experienced shipmates are able to console them by recounting their past experiences.

It is caused by exposure of the eyes to very bright light, such as a tropical sun reflected off the dazzling surface of the tropical ocean, and the history obtained from patients of this class is often, that during the previous part of the voyage they have suffered in health, or been underfed, or have found the ship's food distasteful, which, in addition to the unaccustomed shortness of the periods allotted to slumber and recouping of the energies, have caused an undue strain on the system, and created a general depression of the physical powers of resistance to unsuitable external influences and surroundings.

The same remarks apply also to soldiers campaigning or on long marches who are exposed to the glare of the sunshine, and liable to find rations run short, and to be bereft of their usual and necessary amount of sleep, so that their vitality becomes lowered. This also accounts for the cases recorded accompanying attacks of scurvy.

It is also produced by bright artificial light, such as the electric arc light. It is also seen in a modified degree in persons working many consecutive hours at white work, as seamstresses, sailmakers, whitewashers, painters, and tinworkers do.

When one has been walking in the bright sunlight, and enters a house with a dimly-lit or shaded hall, it is often very difficult to see about one at first; this is a mild manifestation of the same phenomenon, the retina, however, in this case quickly recovers, and adapts itself to the altered circumstances. Here again it also is typical of the more severe cases, as they, when put in suitable surroundings and under suitable treatment, rapidly recover. These cases as a rule are not accompanied by photophobia, thus differing from the form of overstimulation of the retina found in Alpine climbers who have neglected to wear darkened glasses or veils, and which is known as snow-blindness. Night-blindness is the prominent symptom also in retinitis pigmentosa, but in this disease there is also a permanent narrowing of the field of vision, and the ophthalmoscope shows the characteristic patches of retinal pigment, black and somewhat star-shaped, like bone corpuscles. There is sometimes a narrowing of the visual field detected by careful examination in cases of ordinary night-blindness, but it is only temporary in nature and recovers with the patient's other symptoms. Colour vision is often slightly affected, blue and green being confounded. Examination of the eye in these cases of idiopathic night-blindness is almost negative, there is some loss of activity in the movements of the pupil, which is partially dilated, the media are clear, and nothing abnormal is discovered by the ophthalmoscopic examination of the fundus oculi. It is therefore due to an asthenic condition of the retinal cell elements, a nervous exhaustion from over stimulation, in fact a torpor of the retina.

When the unfavourable conditions are removed and replaced by favourable ones—good food, good hygenic surroundings, rest from the stimulation of bright light by wearing darkened glasses, known as London smoked or blue glasses—the vision improves, and the untoward symptoms quickly pass off, leaving the sight in its normal condition. There is a tendency for the symptoms to relapse when placed again under similar circumstances. It is, therefore, advisable to change the patient's work for awhile where possible, and to warn those who cannot change to take extra precautions to protect the eyes from excessive stimulation, and to keep the physical powers up to the best mark possible, and these are the main principles of treatment. The eyes should be shaded during any necessary or unavoidable exposure to bright lights, by shady hats; blue or London smoked glasses should be worn. When gazing at a bright light or an eclipse of the sun use a piece of glass smoked by holding it over a lamp flame for a few seconds, if the coloured glass is not at hand. Rest, good food, and tonics are necessary adjuncts. Iron, strychnine and phosphorus, cod liver oil, &c., should be administered, fresh meat, fruit, and vegetables should be taken when obtainable, and the usual substitutes, as limejuice, when not. Myotics are useful by partially closing the pupil and shutting out excessive light during the stage of convalescence, and allowing the retina to be placed near a state of rest.

Another class of night-blindness in which torpor of the retina is not the cause is the result of a central opacity of the lens in an early stage—not too opaque for bright rays to go through and stimulate the retina. The patient is able to get about in daylight, but when dusk comes on the rays of light are not sufficient to penetrate, and therefore the patient compares much more unfavourably with other people than during daylight. I saw a case of this the other day, where the subject had lost one eye by an accident. He complained of not being able to see after dusk. He was a driver, and able to drive by daylight safely, but at night not only was he handicapped by the dim light, but if he met a vehicle with a bull's-eye or other bright light the rays from it penetrated his eye and stimulated the retina at one point, accentuating, however, the surrounding darkness, and making it difficult for him to steer his course. This case would not be much influenced by treatment; an iridectomy would admit more light, however, for a time.

